# Successful Achilles Tendon Reattachment Using SpeedBridge in Haglund’s Triad Patients

**DOI:** 10.3390/medicina61081445

**Published:** 2025-08-11

**Authors:** Roberto Bevoni, Elena Artioli, Davide Censoni, Marco Di Ponte, Silvio Caravelli, Massimiliano Mosca

**Affiliations:** 1 Orthopaedic Department, IRCCS Istituto Ortopedico Rizzoli, 40010 Bentivoglio, Italy; 2 IRCCS Istituto Ortopedico Rizzoli, 40136 Bologna, Italy; 3 Department of Biomedical and Neuromotor Sciences (DIBINEM), Alma Mater Studiorum University of Bologna, 40123 Bologna, Italy; 4 S.C. Ortopedia e Traumatologia, Ospedale Maggiore “Pizzardi”, 40133 Bologna, Italy

**Keywords:** Achilles tendon, tendinopathy, heel pain, Haglund, SpeedBridge

## Abstract

*Background and Objectives:* The surgical approach for Haglund’s triad remains a topic of ongoing debate. This study presents pioneering data on Achilles tendon reattachment using the SpeedBridge technique following partial detachment, retrocalcaneal bursa excision, and Haglund prominence resection in patients with Haglund’s triad. *Materials and Methods:* A retrospective analysis was conducted on patients operated on between March 2019 and March 2022, encompassing demographic data and preoperative and 6-month and 12-month postoperative assessments of the VAS (Visual Analog Scale), AOFAS (American Orthopedic Foot & Ankle Society) score, and SF-36. *Results:* Nine patients (three females and six males) with a mean age of 53.8 years underwent surgery, with a mean follow-up of 34 months. The results indicated a significant improvement (*p* < 0.0001) in the VAS, AOFAS, and SF-36 at the 12-month follow-up. *Conclusions:* Taking into account the limitations of the study, including its retrospective design and the small sample size, the use of SpeedBridge for Achilles tendon reattachment, alongside the aforementioned procedures, demonstrated promising outcomes. These findings warrant further investigation in future randomized studies with larger sample sizes to confirm their efficacy.

## 1. Introduction

Posterior heel pain represents a common clinical concern, often attributed to various conditions such as Haglund’s deformity, insertional Achilles tendinosis, or retrocalcaneal bursitis [1]. While each of these conditions may manifest independently, they can also coexist, giving rise to a clinical scenario known as Haglund’s triad [1]. Haglund’s deformity was first described by Patrick Haglund in 1927, who observed an evident prominence on the posterolateral aspect of the heel accompanied by tenderness in the lateral region of the Achilles tendon [2]. This deformity commonly results in insertional Achilles tendinopathy and the inflammation of the retrocalcaneal bursa, primarily triggered by the interplay between ankle dorsiflexion and pressure from the shoe against the prominent area [3]. In addition to the evident posterior prominence, Haglund’s triad clinically presents with chronic posterior heel pain and swelling, exacerbated by increased physical activity (e.g., walking, running, stair climbing) chiefly attributable to the insertional Achilles tendinopathy. Furthermore, retrocalcaneal bursitis induces pain upon the palpation of the retrocalcaneal recess, situated just proximal and anterior to the Achilles tendon insertion [4]. Beyond the clinical presentation, radiological examinations play a crucial role in diagnosis. Radiographs allow for the measurement of Fowler’s angle and parallel pitch lines to assess the degree of prominence and identify any intratendinous calcifications [4]. Additionally, magnetic resonance imaging (MRI) can reveal inflammatory insertional changes [5]. The initial therapeutic strategy is conservative and primarily focuses on alleviating pressure and inflammation in the posterior heel region. This involves the use of open-back shoes to mitigate impingement, along with the administration of anti-inflammatory drugs. Moreover, efforts are directed towards reducing Achilles traction stress through the implementation of heel lifts. Complementary interventions include physical therapy, stretching exercises, and eccentric gymnastics. Additional treatments encompass extracorporeal shock wave therapy, local corticosteroid injections, and sclerosing therapy, with outcomes demonstrating variability in reported cases [3]. Conservative treatment is generally successful in a high percentage of patients, with success rates reported up to 85–95%. Consequently, only 5–15% of patients who are refractory to conservative measures eventually undergo surgical intervention [6]. Surgical intervention is generally recommended following the lack of improvement during a conservative treatment period lasting at least six months [7]. At present, there is no consensus on the gold standard for surgical treatment for Haglund’s triad, and both open and endoscopic techniques have been described in the literature, yielding diverse outcomes and complications [8,9,10]. Nonetheless, the primary surgical goal remains retrocalcaneal bursectomy and the excision of the Haglund bony deformity, with or without the debridement and reinsertion of the Achilles tendon. An important concern with surgery involving tendon detachment and reinsertion is the potential risk of tendon rupture or pull-out afterward. For this reason, the method of Achilles tendon reattachment following the surgical treatment of Haglund’s triad becomes particularly crucial [11]. Recently introduced to the market is the “SpeedBridge” anchor system (Arthrex, Inc., Naples, FL, USA) for Achilles tendon reinsertion post the resection of Haglund’s deformity. This system, derived from the widely used “SutureBridge” device for rotator cuff injuries in the shoulder, features an hourglass suture configuration over the distal tendon with a four-anchor setup, eliminating the need for knots [12]. In the context of identifying the optimal surgical solution for managing Haglund’s triad, this study aimed to present a case series of patients who underwent tenolysis, the tangential resection of the posterior calcaneal prominence, and Achilles tendon reinsertion using the Arthrex SpeedBridgeTM System (Arthrex, Inc., Naples, FL, USA). The assessment concentrated on evaluating the validity and reproducibility of the system, as well as analyzing the resultant impact on the quality of life of the patients.

## 2. Materials and Methods

### 2.1. Patients and Study Design

This study was approved by the local Ethics Committee under protocol number 144/2025/Oss/IOR.

A retrospective observational study was conducted on consecutive patients who underwent surgical treatment for Haglund’s triad involving a posterior central Achilles tendon splitting approach, partial tendon detachment, tendon debridement, the excision of the retrocalcaneal bursa, Haglund prominence resection, and Achilles tendon reattachment with the SpeedBridge anchor system. The study included cases treated operatively from March 2019 to March 2022.

**Figure 1:**
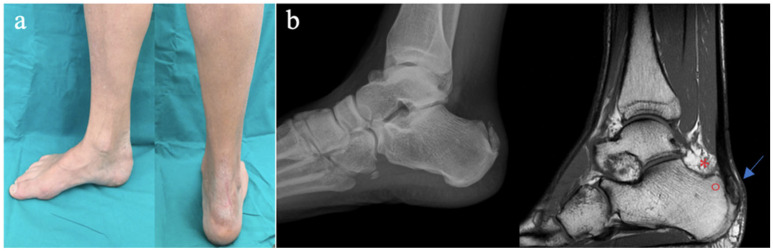
(**a**) Preoperative clinical images of Haglund’s triad; (**b**) preoperative radiographical images of Haglund’s triad showing Haglund’s deformity (°), insertional Achilles tendinosis (arrow), or retrocalcaneal bursitis (*).

Patients were included according to the following criteria: (1) age greater than 18 years, (2) patients who provided their consent to participate in the study, (3) patients capable of adhering to postoperative instructions, (4) clinical and radiological evidence of Haglund’s deformity (Figure 1a), (5) clinical and radiological evidence (ultrasound or MRI) of insertional calcific tendinopathy and retrocalcaneal bursitis (Figure 1b), and (6) an intraoperative diagnosis of Haglund’s triad.

Exclusion criteria were as follows:

- A history of prior Achilles tendon rupture;
- Previous surgery on the same heel;
- Treatment performed with a different surgical technique;
- Incomplete follow-up;
- Age under 18 years;
- A lack of informed consent to participate in the study;
- An inability to comply with postoperative care instructions.

All surgical procedures were performed by a senior orthopedic surgeon specialized in foot and ankle conditions.

### 2.2. Data Collection

This study gathered patients’ demographic information, including age, gender, and body mass index, along with operative data such as surgical time and complications, through a retrospective data collection approach. Postoperative outcomes at both 6 and 12 months were also assessed retrospectively. Prior to surgery and during the postoperative period, patients were requested to complete outcome questionnaires. Clinical outcome measures were collected using the Visual Analog Scale (VAS), the American Orthopedic Foot and Ankle Society (AOFAS) Ankle and Hindfoot Scale, the Short Form-36 (SF-36), and a satisfaction survey. This comprehensive approach allowed for a thorough evaluation of patient-reported outcomes and clinical assessments at 6 and 12 months postoperatively.

Pain was quantified using a VAS with scores ranging from 0 (indicating no pain) to 10 (representing maximal imaginable pain) [13]. The assessment of functional level, both pre- and postoperatively, utilized the AOFAS Ankle and Hindfoot Scale, which assigns a total of 100 points graded for Pain (40 points), Function (50 points), and Alignment (10) [14]. The SF-36 of RAND Health Survey 1.0 was employed as a general health scale, providing physical (PCS) and mental component summary (MCS) scores. It comprises 36 items distributed across 8 subscales [15]. Furthermore, patients were asked to rate their overall subjective satisfaction after surgery during their last follow-up visit, utilizing a rating scale with categories ranging from excellent, very good, good, moderate, poor, and terrible.

Complications were classified according to the Modified Clavien-Dindo-Sink classification [16], which includes the following grades:

- Grade I: Adverse events with minimal clinical relevance that do not cause deviation from routine postoperative follow-up. Therapeutic measures include analgesia, antibiotics, and physiotherapy.
- Grade II: Complications that are treatable but do not require additional surgical intervention or unplanned hospital admission.
- Grade III: Complications that are treatable and require surgical intervention or unplanned hospital admission.

### 2.3. Surgical Technique

**Figure 2:**
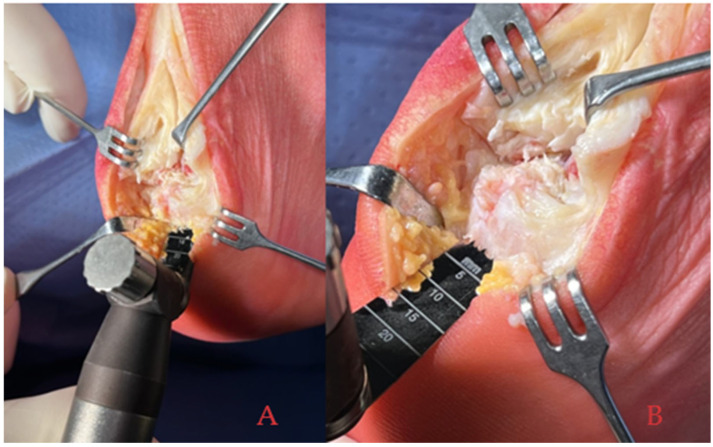
(**A**) Posterior view of Haglund’s prominence excision using oscillating saw. (**B**) Lateral view of Haglund’s prominence excision using oscillating saw.

The patient was placed in a prone position and a tourniquet was applied. The posterior central Achilles tendon split approach, as described by McGarvey et al. [17], was performed in all patients. This approach was preferred because it provides excellent exposure, minimizes the risk of sural nerve injury, and preserves the vascular supply to the Achilles tendon. A partial tendon detachment was made from the midline to the lateral aspect of the calcaneus, both medially and laterally. This method ensured sufficient exposure for the meticulous debridement, tenolysis, and excision of the calcifications while maintaining the connection of the tendon without complete detachment. Subsequently, the retrocalcaneal bursa was excised to unveil the superior aspect of the calcaneus and Haglund’s prominence. The latter was then resected using an oscillating saw and refined with a Luer-type rongeur (Figure 2).

**Figure 3:**
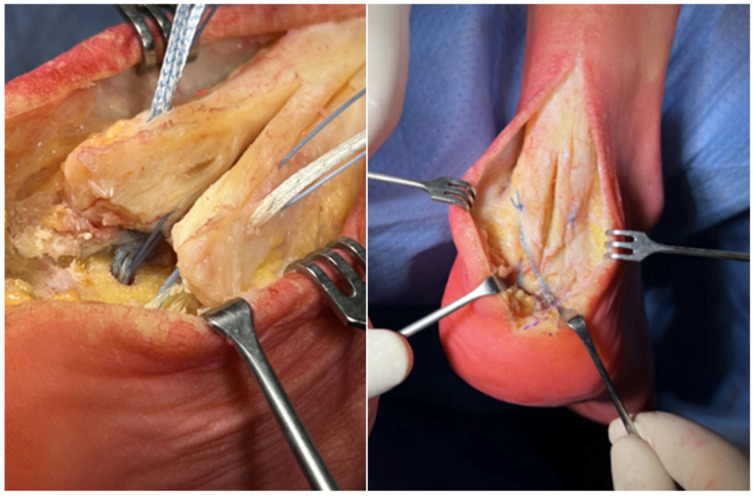
The placement of FiberTape sutures across the Achilles tendon and the hourglass configuration of the construct.

Following the management of all components of Haglund’s triad, the Achilles tendon was reattached to its calcaneal insertion using two 4.75 mm SwiveLock Anchors (Arthrex, Naples, FL, USA) loaded with 2 FiberTape sutures (Arthrex, Naples, FL, USA) proximally. These anchors were then connected to two additional SwiveLock Anchors distally, creating an hourglass configuration following the SpeedBridge technique (Figure 3). Throughout the procedure, the foot was kept in a plantigrade position to minimize stress on the construct.

The longitudinal incision along the tendon fibers was repaired using 0 Vicryl sutures. Subsequently, peritenon repair was performed with 2-0 Vicryl sutures, ensuring inward-facing knots. The skin closure was achieved using 3-0 Prolene sutures.

**Figure 4:**
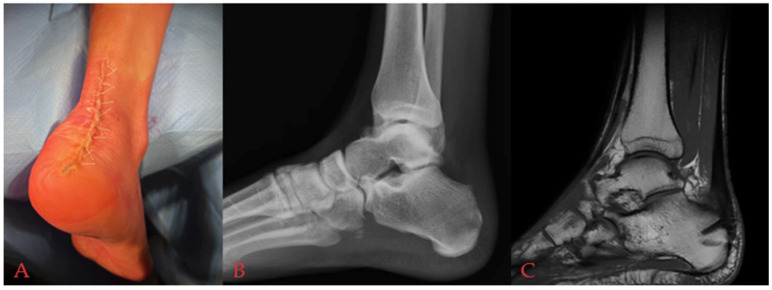
(**A**) Postoperative clinical image. (**B**) Postoperative X-rays. (**C**) Postoperative MRI.

Post-surgical radiological assessment was taken to evaluate the posterior aspect of the calcaneus. Standard lateral radiographs were obtained intraoperatively in the operating room. MRI was sometimes requested at a minimum of 6 or 12 months postoperatively to assess soft tissue healing and residual inflammation (Figure 4). A Walker-type boot, equipped with a removable 10° wedge to achieve a gravitational equinus of the foot, was permanently applied to all patients. All patients were discharged on the first postoperative day, with a prescribed period of partial weight-bearing using crutches for the initial 4 weeks post surgery. Following this, the removal of the wedge and progressive weight-bearing within the Walker boot was advised. By the 6th postoperative week, walking aids were discontinued, allowing patients to transition to comfortable footwear. Hence, all patients were given rehabilitation instructions consisting of repeated Achilles tendon and ankle stretches, range of motion exercises, and proprioception training to be continued for at least 2 months.

### 2.4. Statistical Analysis

Statistical analysis was performed using SPSS, version 19 (IBM Corp., Armonk, NY, USA). Continuous variables resulting from clinical scores were reported as mean values, standard deviation (SD), and range, whereas categorical variables were described as frequency and percentage. The data distribution was tested for normality through the Shapiro–Wilk test. It was assumed that the data from the various groups were independent and that there was a homogeneity of variances. After verifying the normality of the data, the paired *t*-test was used to evaluate differences in preoperative and postoperative data outcomes. The statistical significance was set at *p*-value < 0.05.

## 3. Results

**Figure 5:**
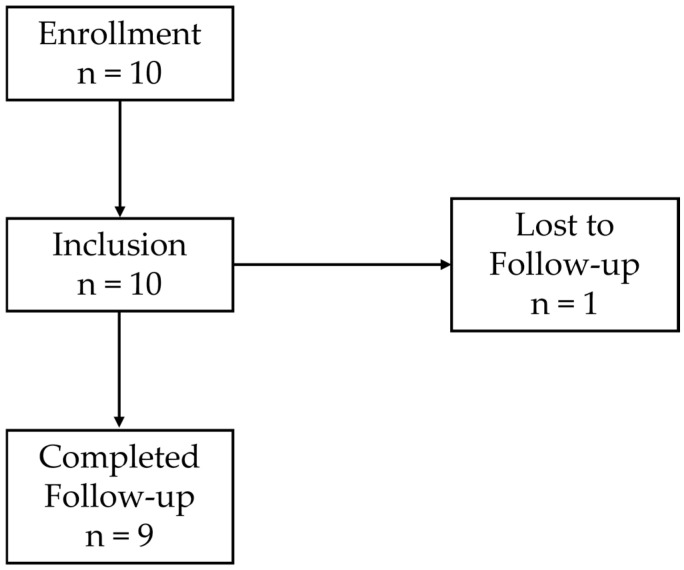
Flowchart of patient selection and follow-up process.

Ten consecutive patients (ten heels) diagnosed with symptomatic Haglund’s triad underwent surgical correction involving a posterior central Achilles tendon splitting approach, partial tendon detachment, tendon debridement, the excision of the retrocalcaneal bursa, Haglund prominence resection, and Achilles tendon reattachment with the SpeedBridge anchor system between March 2019 and July 2022. Out of the initial ten patients, one was lost to follow-up, resulting in nine patients (nine heels) available for chart review. All patients met the inclusion criteria and were included in this consecutive case series (Figure 5).

### 3.1. Population

Among the nine patients, three were female (33.3%) and six were male (66.7%). The mean age at surgery was 53.8 ± 8.6 years (range, 42–67). The mean body mass index (BMI) was 24.2 ± 5.4 kg/m^2^ (range, 18.6–33.6). Among the nine feet, three were right (33.3%) and six left (66.7%). The average follow-up was 34.0 ± 14.1 months (range, 12–48). The mean surgical time was 58.2 ± 10.9 min (range, 40–72). Patients’ demographics are reported in Table 1.

### 3.2. Clinical Outcomes

**Figure 6:**
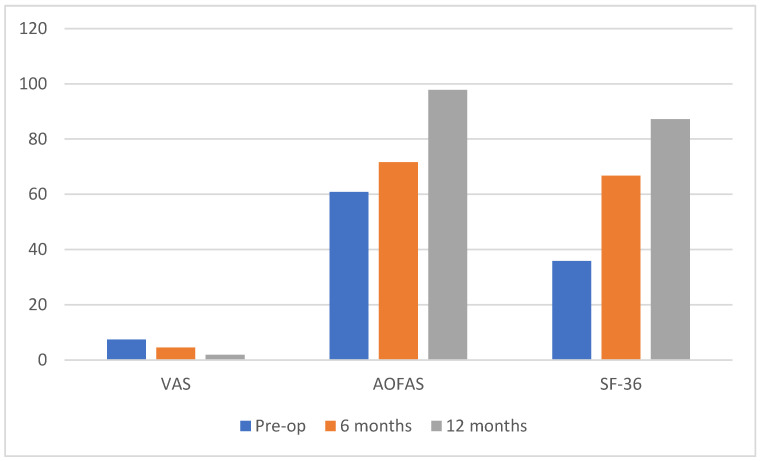
Graph illustrating the trends in VAS, AOFAS and SF-36 scores.

The mean VAS pain score showed a significant improvement from 7.4 ± 0.5 preoperatively to 4.5 ± 1.4 at 6 months and further decreased to 1.9 ± 1.2 at 12 months postoperatively. Concurrently, the mean ankle–hindfoot AOFAS score demonstrated a significant increase from 60.8 ± 9.7 preoperatively to 71.6 ± 13.4 at 6 months and further improved to 97.8 ± 5.3 at 12 months. Similarly, the mean SF-36 exhibited a significant enhancement from 35.8 ± 3.8 preoperatively to 66.7 ± 8.9 at 6 months and rose to 87.2 ± 8.5 at 12 months. All these improvements were statistically significant, with a *p*-value < 0.0001 for all scores analyzed, both between preoperative and 6-month assessments and between 6-month and 12-month assessments. A comprehensive summary of the results is presented in Table 2 and Figure 6.

Patient satisfaction with the surgical intervention was evaluated, with 22% rating their satisfaction as excellent, 33% as very good, and an additional 33% as good. A smaller proportion, 11% (one patient), rated his satisfaction as fair. In aggregate, the overall subjective satisfaction rate ranging from good to excellent was 88%, representing eight out of nine patients.

Postoperative complications were limited, with only one case of delayed superficial wound healing, successfully addressed through dressing changes and a superficial debridement (Grade I according to the modified Clavien–Dindo–Sink classification [16]).

No occurrences of complications, such as Achilles tendon ruptures, infections, or painful scars, were recorded.

## 4. Discussion

This study evaluated the outcomes of surgical correction for Haglund’s triad through a posterior central Achilles tendon splitting approach with partial tendon detachment, retrocalcaneal bursa excision, tendon debridement, Haglund’s prominence resection, and Achilles tendon reattachment using the SpeedBridge anchor system.

The effectiveness of treatment through retrocalcaneal bursa excision, adequate Achilles tendon debridement, and Haglund’s prominence resection for Haglund’s triad has been previously demonstrated in the literature [18]. For instance, Miao et al. analyzed 34 patients, observing a significant improvement in the mean VAS score, decreasing from 6.5 preoperatively to 2.1 at mean follow-up of 45.2 ± 17.7 months [19]. Similarly, Ettinger et al. examined 40 patients, noting a significant improvement in VAS scores from 8.5 preoperatively to 2.6 postoperatively, alongside a noteworthy increase in the mean ankle and hindfoot AOFAS score from 59.4 to 86.5, at a mean follow-up 15.6 ± 3.7 months [20]. Furthermore, Ahn et al. reported an increase in mean ankle and hindfoot AOFAS scores from 62.1 preoperatively to 92.5 postoperatively in their study involving 15 patients at a mean follow-up of 3.5 ± 1.5 years [21].

Perioperative quality of life, assessed using the SF-36 score, was examined by Anderson et al., who reported an improvement in the PCS subcategory [22], while Ettinger et al. documented significant improvements in the physical, pain, and mental subscales [20].

Patients in this study reported favorable pain relief, functional outcomes, and quality of life, with results comparable to previously published studies. Although the improvement in clinical scores over time reached statistical significance, it should be interpreted with caution. Given the small sample size and the multifactorial nature of recovery—including rehabilitation protocols and natural progression—this finding may not directly reflect the isolated effect of surgical intervention. Regardless, the overall postoperative satisfaction rate in the present study was 88%, which aligns with rates reported in the literature ranging from 75% to 100% [4,23]. Therefore, this study provided further confirmation of the effectiveness of surgical treatment including retrocalcaneal bursa excision, Achilles tendon debridement, and prominence resection, in managing Haglund’s triad.

Various surgical techniques have been described for performing this treatment, involving both endoscopic and open approaches [24]. The endoscopic approach, while providing advantages, proved to be more challenging in addressing Achilles tendon calcifications and performing meticulous degenerated tissue debridement, with a long learning curve required [20,25]. Therefore, an open approach has traditionally been favored for the surgical treatment of Haglund’s triad [18].

Open approaches can be executed through different incisions, including longitudinal incisions (such as posteromedial, posterolateral, and central tendon splits), J-incision, or a combination of posteromedial and posterolateral incisions [4]. In this study, the central tendon splitting approach was employed, as described by McGarvey et al. who found that patients exhibited central Achilles tendinopathy in 95% of cases, with only 14% involving the lateral tissue, either additionally or separately [17]. This approach offers the advantage of excellent exposure, allowing the surgeon to address all pathological elements in the posterior part of the heel without involving the medial and lateral edges of the Achilles tendon. This helps avoid the risk of sural nerve injury, minimizes impairment to the vascular supply of the Achilles tendon, and facilitates any tendon lengthening [17,19,26,27]. Moreover, a cadaveric study showed an additional benefit, with the incision being placed along the peroneal and posterior tibial angiosomes [28]. Furthermore, patients undergoing surgery via the central trans-tendon approach were reported to return to normal function earlier than those treated with the posterolateral approach [22].

A reported drawback of this approach is the potential for several postoperative complications, particularly a painful scar due to shoe irritation [17,28,29,30]. No such issue was observed, and no major complications occurred in this study, with only one wound-related complication. This aligns with the literature findings since a recent systematic review reported the incidence of wound complications with the central tendon split approach (7.0%) as no greater than that with the posteromedial approach (8.3%) reported in other articles [28,30].

Once the surgical approach is defined, the detachment of the Achilles tendon, either partially or completely, becomes necessary to reach adequate tendon debridement and remove the Haglund’s prominence. However, it is crucial to consider that the extent of tendon detachment is correlated to its risk of rupture. A biomechanical cadaveric study demonstrated that Achilles tendons could be detached up to 50% without a risk of rupture [11]. In addition, Calder and Saxby assessed that a less than 50% detachment of the Achilles tendon allowed safe early postoperative active mobilization without risking rupture [31].

Moreover, the complete detachment of the Achilles tendon could potentially impair ankle plantar flexion strength. Wagner et al. observed a 4% loss of plantar flexion strength in patients with insertional Achilles tendinosis who had undergone the complete detachment of the Achilles tendon, associated with a proximal V-Y lengthening and reinsertion of the tendon [32]. Therefore, partial detachment is recommended, and reinsertion should be considered to prevent complete rupture and restore ankle plantar flexion strength.

Many techniques have been described for Achilles tendon reinsertion, but the optimal technique remains controversial [18,33,34,35]. Jiang et al. and Ettinger et al., as early as 2016, demonstrated that the double-row suture technique yields superior results compared to the single row at a follow-up time similar to this study [20,36]. However, in the context of Haglund’s triad, the use of the SpeedBridge technique has been minimally explored in the literature. Kar et al. demonstrated excellent improvement in the AOFAS ankle–hindfoot score in 13 patients at 24 months of follow-up, even allowing early postoperative weight bearing and mobilization [37]. Similarly, Lewis et al., in their study of 50 patients evaluated at a mean follow-up of 2.4 ± 1.9 years, treated with a double-row suture bridge technique for insertional Achilles tendinopathy, reported that the method is both safe and effective, with no tendon rupture and only four minor complications that did not affect the normal clinical course [38].

Guler et al. have confirmed the effectiveness and safety of the treatment for Haglund syndrome at a mean follow-up period of 30 months, utilizing a similar reconstruction technique [39]. Witt and Hyer reviewed data from four Haglund triads that underwent complete Achilles tendon detachment and reinsertion with four suture anchors, reporting no tendon ruptures during their follow-up period of approximately 2 years [18].

In this study, employing a similar reinsertion technique, none of the patients experienced Achilles tendon rupture over an average follow-up of 34 months. No patient required reoperation, and only a minor complication, which did not impact the clinical course, was observed. Furthermore, the reinsertion of the tendon using the SpeedBridge anchor system, with the preservation of most of the original tendon attachment, may help reduce the risk of plantar flexion strength loss otherwise associated with the complete detachment of the Achilles tendon, as reported by Wagner [32]. This study thus aligns with evidence supporting the effectiveness of the SpeedBridge anchor system [18,20,36,37,38,39]. While the SpeedBridge anchor system is notably more expensive than the traditional single anchor, a cost-effectiveness study is warranted. To date, only one study has evaluated this, concluding that double-row fixation is more costly and does not offer clinical advantages over the single anchor [40]. However, the revision rate for the single anchor was double that of the double row (6.4% vs. 3.1%), and complications were also higher (9% vs. 6.3%), although not statistically significant. However, revisions can significantly influence the overall total costs. Moreover, no power analysis was performed to determine the sample size required to achieve statistical significance. Additionally, the study sample size was uneven between groups (78 with a single anchor repair and 32 with a double row), which could have introduced bias. Thus, the lack of significant findings may be attributed to a lack of statistical power [40].

This study has some limitations. Firstly, being a non-comparative retrospective study, it can demonstrate outcomes of the SpeedBridge technique but cannot highlight any superiority over other techniques. Secondly, the number of patients is very limited, with only nine included, due to the strict inclusion criteria, though this is consistent with other published paper on this topic.

Further prospective multicenter randomized studies with a more extensive sample size and a longer follow-up would be necessary to comprehensively evaluate the outcomes of this Haglund’s triad surgery and potential associated risk factors. However, the strengths of this study lie in the methodical approach to patient follow-up and data collection. All presented data were gathered at specific time points for each patient, ensuring complete data availability across the entire cohort. Furthermore, both joint-specific and non-joint-specific Patient-Reported Outcome Measures (PROMs) were utilized, allowing a clear presentation of patients’ conditions and enabling comparisons with other techniques. Finally, to our knowledge, this case series remains one of the longest follow-ups in the literature regarding the use of the SpeedBridge technique in the context of Haglund’s triad.

## 5. Conclusions

The results of this study demonstrated that the surgical correction of Haglund’s triad through the posterior central Achilles tendon splitting approach, partial tendon detachment, tendon debridement, the excision of the retrocalcaneal bursa, Haglund prominence resection, and Achilles tendon reattachment using the SpeedBridge device proved to be an effective treatment. The procedure resulted in significant pain relief and functional improvement, coupled with a minimal complication rate over a mid-term follow-up period. However, to fully validate these promising findings and determine their generalizability, there is a clear need for larger, prospective studies. Such studies would not only confirm the efficacy and safety of this approach but also provide insights into cost-effectiveness analysis and optimal patient selection and refine the treatment protocol.

## Figures and Tables

**Table 1:** Patients’ demographic details. M: male; F: female; R: right; L: left; SD: standard deviation BMI: body mass index.

	Mean	SD	Range
**Age (years)**	53.8	8.6	42–67
**Gender**	3 M–6 F		
**BMI (kg/m^2^)**	24.2	5.4	18.6–33.6
**Side**	3 R–6 L		
**Surgical Time (minutes)**	58	11	40–72
**Follow-up (months)**	34.0	14.1	12–48

**Table 2:** Clinical outcomes–mean values and standard deviations.

	Pre-op	6 Months	12 Months	*p*-Value
**VAS**	7.4 ± 0.5	4.5 ± 1.4	1.9 ± 1.2	*p* < 0.0001
**AOFAS**	60.8 ± 9.7	71.6 ± 13.4	97.8 ± 5.3	*p* < 0.0001
**SF-36**	35.8 ± 3.8	66.7 ± 8.9	87.2 ± 8.5	*p* < 0.0001

## Data Availability

The data presented in this study are available on request from the corresponding author due to privacy, legal and ethical reasons.
